# Fast automated cell phenotype image classification

**DOI:** 10.1186/1471-2105-8-110

**Published:** 2007-03-30

**Authors:** Nicholas A Hamilton, Radosav S Pantelic, Kelly Hanson, Rohan D Teasdale

**Affiliations:** 1 ARC Centre in Bioinformatics, University of Queensland, Brisbane, Queensland 4072, Australia; 2 Institute for Molecular Bioscience, University of Queensland, Brisbane, Queensland 4072, Australia; 3 Advanced Computational Modelling Centre, University of Queensland, Brisbane, Queensland 4072, Australia

## Abstract

**Background:**

The genomic revolution has led to rapid growth in sequencing of genes and proteins, and attention is now turning to the function of the encoded proteins. In this respect, microscope imaging of a protein's sub-cellular localisation is proving invaluable, and recent advances in automated fluorescent microscopy allow protein localisations to be imaged in high throughput. Hence there is a need for large scale automated computational techniques to efficiently quantify, distinguish and classify sub-cellular images. While image statistics have proved highly successful in distinguishing localisation, commonly used measures suffer from being relatively slow to compute, and often require cells to be individually selected from experimental images, thus limiting both throughput and the range of potential applications. Here we introduce *threshold adjacency statistics*, the essence which is to threshold the image and to count the number of above threshold pixels with a given number of above threshold pixels adjacent. These novel measures are shown to distinguish and classify images of distinct sub-cellular localization with high speed and accuracy without image cropping.

**Results:**

Threshold adjacency statistics are applied to classification of protein sub-cellular localization images. They are tested on two image sets (available for download), one for which fluorescently tagged proteins are endogenously expressed in 10 sub-cellular locations, and another for which proteins are transfected into 11 locations. For each image set, a support vector machine was trained and tested. Classification accuracies of 94.4% and 86.6% are obtained on the endogenous and transfected sets, respectively. Threshold adjacency statistics are found to provide comparable or higher accuracy than other commonly used statistics while being an order of magnitude faster to calculate. Further, threshold adjacency statistics in combination with Haralick measures give accuracies of 98.2% and 93.2% on the endogenous and transfected sets, respectively.

**Conclusion:**

Threshold adjacency statistics have the potential to greatly extend the scale and range of applications of image statistics in computational image analysis. They remove the need for cropping of individual cells from images, and are an order of magnitude faster to calculate than other commonly used statistics while providing comparable or better classification accuracy, both essential requirements for application to large-scale approaches.

## Background

Obtaining the sequence of numerous genomes and subsequent identification of the encoded proteome has created the need for large-scale systematic approaches to understand the functions of the tens of thousands of proteins at the cellular level [1,2]. High-throughput automated fluorescent microscope imaging technologies enable the experimental determination of a protein's sub-cellular localization and its dynamic trafficking within a range of cellular contexts. These approaches generate vast numbers of images including multiple fluorophores for cells under a variety of experimental conditions [3,4]. Furthermore, cells may now be imaged in 3D, or indeed 4D with 3D stacks captured over time to observe protein trafficking in live cells [5]. The desire and the ability to carry out high-throughput screenings of protein localization and trafficking for applications such as drug discovery [4] is leading to a rapid growth in cell images in need of analysis on a scale comparable to that of the genomic revolution. It has been estimated that to take a single image for each combination of protein, cell type and timescale would require of the order of 100 billion images [6]. Currently, image databases such as the *Yeast GFP Fusion Localization Database *[7], the *LOCATE mouse protein sub-cellular localization database *[2] and the *LIFEdb database *for the integration and dissemination of functional data [8] offer the possibility to present, integrate and search the vast amounts of data being created by high throughput cell imaging. However, to a large degree the analysis and comparison of localizations are still performed by the slow, coarse-grained and possibly biased process of manual inspection. To deal with the scale of the data becoming available automated annotation, analysis, comparison, classification and storage of cellular images is essential.

Image statistics have proven to be of great utility in the automated analysis of cellular images. Haralick texture measures define a variety of statistics based on the spatial dependence of individual pixel intensities across an image [9]. Zernike moments [10,11] calculate the decomposition of an image onto an orthogonal set of polynomials in much the same way that Fourier coefficients may be used to decompose a time series. These and other [12] measures may be used to generate a vector of numbers for a given cell image, and have a wide range of applications. For a given set of images, a representative image may be chosen by selecting the image with vector closest to the mean vector of the entire image set [13,14]. Images may be clustered [15] or ranked by distance from a given image to find similar images [14]. And given two sets of sub-cellular localization images under differing experimental conditions, image statistics can be used to assess whether there is a statistically significant difference, even to the extent that visually indistinguishable images of distinct localizations may be differentiated [16]. One important application is in automated sub-cellular localization classification. Here, a machine learning technique such as a neural network [17] or support vector machine (SVM) [18] is trained on image vectors of known localization, and subsequently used to predict those of unknown localization. With accuracies of well over 90% [19-21] such predictors have proved very successful, and have exceeded human classification accuracy [13,19]. Once statistical techniques such as the above are fully integrated into cell image databases, a much greater degree of refinement, content searching, unbiased clustering and hypothesis testing will be enabled.

While image statistics have performed well in sub-cellular localization classification, they often suffer from high computational cost and require individual cells to be cropped from an image, hence limiting the extent to which they may be applied. Here, we introduce *threshold adjacency statistics (TAS)*, a simple and fast morphological measure for distinguishing sub-cellular localization.

## Results and discussion

### Algorithm

**Figure 1:**
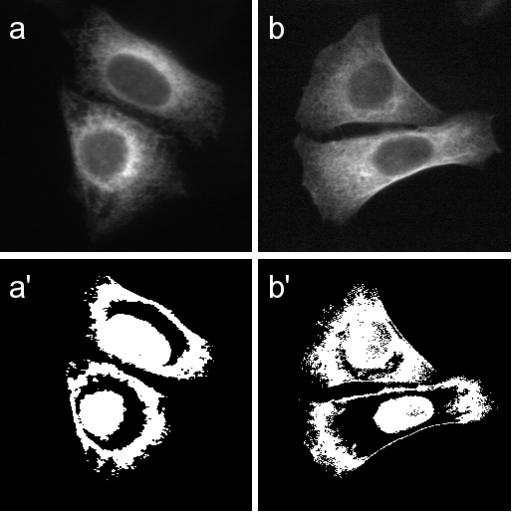
**Distinguishing cell images by thresholding**. Images of the endoplasmic reticulum (a) and the microtubule cytoskeleton (b) are thresholded (a' and b') such that pixels with intensity in the range μ-30 to μ+30 are shown in white, where μ is the average pixel intensity of each image. Though images (a) and (b) are texturally and visually similar, images (a') and (b') are more distinguished. Image (a') contains more solid white regions, while (b') shows more interior speckling and feathering of edges.

**Figure 2:**
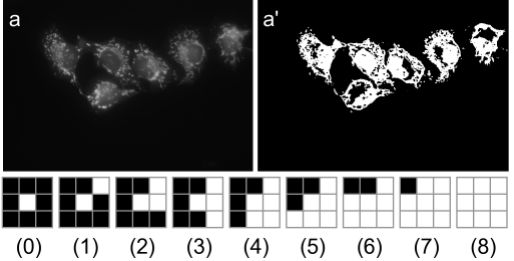
**Threshold statistics for cell images**. Once a cellular image (a) is thresholded (a'), statistics are calculated from the threshold image. For each white pixel the number of pixels adjacent that are also white are counted. Examples of having zero to eight white neighbours are given in (0)-(8). The first threshold statistic is then the number of white pixels with zero white neighbours, the second is the number with one white neighbour, and so on up to eight. These nine statistics are then normalised by dividing each by the total number of white pixels in the threshold image.

Threshold adjacency statistics are generated by first applying a threshold to the image to create a binary image (Figures 1 and 2), with a threshold chosen as follows. The average intensity, μ, of those pixels with intensity at least 30 is calculated for the image, the cut off 30 chosen as intensities below this value are in general background, and is considered in more detail below in the Testing subsection (an 8-bit grayscale image has pixel has intensities from 0 to 255). The experimental image is then binary thresholded to the range μ-30 to μ+30 (Figure 2a'). The range was selected to maximise the visual difference of threshold images for which the localisation images had distinct localisations but were visually similar, as in Figure 1. The following nine statistics were designed to exploit the dissimilarity seen in the threshold images. For each white pixel, the number of adjacent white pixels is counted (Figure 2 (0)-(8)). The first threshold statistic is then the number of white pixels with no white neighbours; the second is the number with one white neighbour, and so forth up to the maximum of eight. The nine statistics are normalised by dividing each by the total number of white pixels in the threshold image. Two other sets of threshold adjacency statistics are also calculated as above, but for binary threshold images with pixels in the ranges μ-30 to 255 and μ to 255, giving in total 27 statistics. A variety of other thresholds ranges were tested but found to give lower performance in later classification tests (data not shown).

### Testing

**Figure 3:**
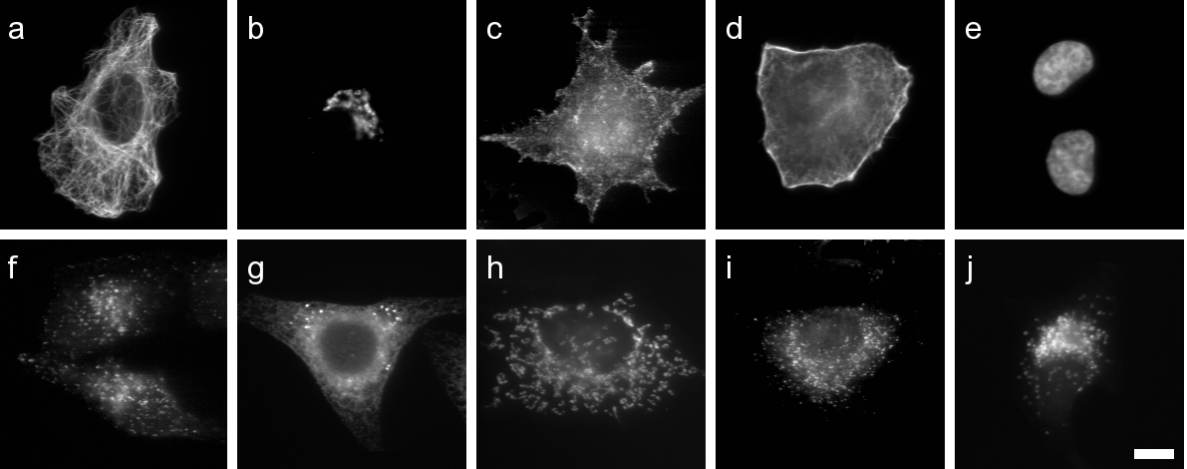
**Sample images of the 10 localisation classes of endogenously expressed proteins**. (a) Microtubule, (b) Golgi, (c) Plasma membrane, (d) Actin cytoskeleton, (e) Nucleus, (f) Endosome, (g) ER, (h) Mitochondria, (i) Peroxisome, (j) Lysosome. Scale bar 10 μm.

**Figure 4:**
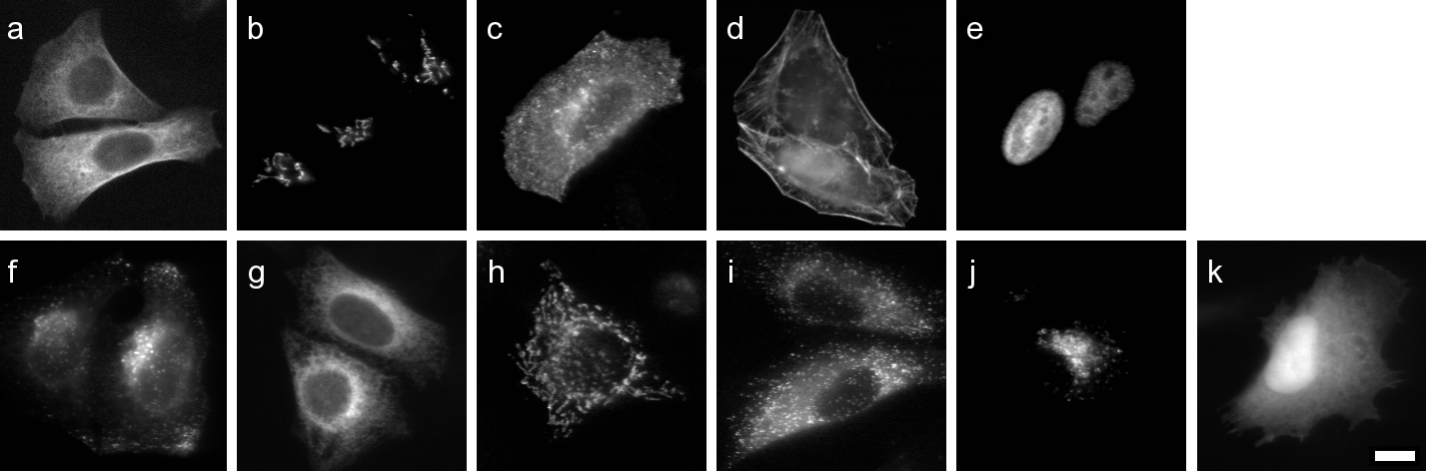
**Sample images of the 11 localisation classes of transfected proteins**. (a) Microtubule, (b) Golgi, (c) Plasma membrane, (d) Actin cytoskeleton, (e) Nucleus, (f) Endosome, (g) ER, (h) Mitochondria, (i) Peroxisome, (j) Lysosome, (k) Cytoplasm. Scale bar 10 μm.

To test the utility of threshold adjacency statistics, we created two epi-fluorescent image collections, each with approximately 50 images per sub-cellular location. One for which an endogenous protein or feature of the specific organelle was detected with a fluorescent antibody or other probe (10 organelles, 503 images). The second a set for which an epitope- or GFP-tagged protein was transiently expressed in the specific organelle and subsequently detected (11 organelles, 553 images). Each image contained between 1 and 13 cells. Details of markers used are given in Table 1, and (cropped) sample images are shown in Figures 3 and 4. The efficacy of threshold adjacency statistics in predicting sub-cellular localization was then tested by generating statistics for the endogenous and transfected images, and creating a SVM for each. For the endogenous set, a 5-fold cross validation classification accuracy (percentage of true positives) of 95.2% was obtained, while the cross validation accuracy on the transfected set was 88.8%. To ensure against potential data bias in cross validation, each data set was randomly split (class-balanced) into 4/5^ths ^for training and 1/5^th ^for testing. A SVM was then trained on the training set and the overall and by localization class classification accuracies on the test set were recorded. Random data splitting, training and testing was then repeated 1000 times. The overall average classification accuracy on the 1000 endogenous test sets was then 94.4%, and 86.3% for the transfected test sets. The classification accuracies for each class of localization for the endogenous set were all high being in the range 92.8% to 98.2%, while the transfected set accuracies showed a wider variation ranging from 76.2% to 95.7% (Table 1). The classes with lower classification accuracy such as mitochondria, ER and plasma membrane appear to be those that exhibit higher visual similarity to each other than to other classes.

**Table 1:** Average classification accuracies using TAS statistics on Endogenous and Transfected data test sets and the subcellular markers used

Organelle	Endogenous Accuracy	Transfected Accuracy	Endogenous Marker	Transfected Marker
Nucleus	97.4%	92.6%	DAPI	myc-nbp-45 (txreg I920050F21)
Cytoplasm	-	95.5%	-	GFP-V5
Endoplasmic Reticulum	90.8%	82.5%	anti-PDI	ICAT-GFP
Golgi	98.2%	87.7%	anti-Beta-COP	GCC-GFP
Plasma Membrane	93.3%	82.4%	anti-EGFR	myc-Lysophosphatidic acid receptor
Endosome	93.9%	88.1%	anti-SNX1	rab5a-GFP
Lysosome	96.5%	76.5%	Lysotracker	myc-chloride channel 7 (5330412O18)
Peroxisome	95.9%	95.7%	anti-catalase	ALD
Mitochondria	92.6%	76.2%	Mitotracker	myc-carntine/acylcarnitine translocase
Actin Cytoskeleton	97.8%	92.3%	Phalloidin	YFP-actin
Microtubules	92.8%	92.1%	anti alpha-tubulin	YFP-tubulin

The accuracies (percentage of true positive predictions) are averaged over 1000 random division of each data set into 4/5^ths ^for training and 1/5^th ^for testing. The overall average classification accuracies on the test sets were 94.4% and 86.3% on the endogenous and transfected sets, respectively.

Both the Haralick texture measures and the magnitudes of the Zernike moments have previously been shown to be useful in distinguishing sub-cellular localization [12,13]. To compare performance with that of threshold adjacency statistics, a set of 20 Haralick measures and 49 Zernike measures were selected. The Haralick measures were chosen from a list of those shown to be good for distinguishing sub-cellular localization in Conrad et al. [12], and have previously been described and tested in the Automated Sub-Cellular Phenotype Classification (ASPiC) system [20]. The Zernike measures chosen were the magnitudes associated with the first 12 Zernike polynomials, and have also previously been applied to sub-cellular localization [13].

Zernike measures require single cell images, and hence the ASPiC automated cropping system was used to select cells from the image. This gave 1420 cell images from the endogenous image set, and 1075 from the transfected set. Haralick, Zernike and threshold adjacency statistics were then generated for each cropped image, and each class of statistics tested using a SVM and 5-fold cross validation. On the cropped endogenous image set, threshold adjacency statistics had a predictive accuracy of 94.4%, the Haralick statistics gave 94.2%, and the Zernike moments gave 75.8% (see Table 2). For the cropped transfected image set, threshold adjacency statistics gave an accuracy of 90.3%, Haralick statistics 86.0%, while Zernike moments gave 68.6%. Hence threshold adjacency statistics had comparable or better results in all cases, with the Zernike moments having significantly lower accuracy than the other two types of image measure. There is a clear trend for the three types of statistics tested to give lower predictive accuracy on the transfected data set.

**Table 2:** Comparison of TAS, Haralick and Zernike statistics classification accuracies by 5-fold cross validation

Image Set	TAS (uncropped)	TAS	Haralick	Zernike	TAS+Haralick
Endogenous	95.2%	94.4%	94.2%	75.8%	98.2%
Transfected	88.8%	90.3%	86.0%	68.6%	93.2%

The 5-fold classification accuracies (as described in Implementation) are shown for each of type of statistic tested. The first TAS column shows accuracies obtained on un-cropped the endogenous (502 images, 10 localisations) and transfected (553 images, 11 localisations) data sets. The remaining columns refer to when individual cells were been selected from the endogenous and transfected data sets, giving 1420 and 1075 cell images, respectively. The 5-fold classification accuracies obtained on the cell selected images using each of TAS, Haralick, Zernike or TAS combined with Haralick are then shown.

To test if the information contained within the threshold adjacency statistics and the Haralick texture measures was complementary, SVMs were trained that combined both types of statistics for each cropped image. In this case, 5-fold cross validated accuracies of 98.2% and 93.2% were obtained on the endogenous and transfected sets, respectively, showing a significant improvement over either individual class of statistic.

The high predictive accuracy when applying Haralick and Zernike statistics comes with the expense of relatively high computational complexity. To compare the computational cost of Haralick, Zernike and threshold adjacency statistics the time taken to calculate each was recorded for the endogenous data set of 503 images. Since the Haralick measures are usually applied to single cell images and Zernike measures require them, the time to crop the 503 images to create 1420 single cell images was first benchmarked and found to be 4 minutes 16 seconds. Generation of 20 Haralick measures then took 11 minutes 50 seconds, and 49 Zernike moments took 17 minutes 22 seconds. This compares to 62 seconds to generate threshold adjacency statistics directly with no cropping for the 503 images. More detailed timing of just the function call to calculate the 27 threshold statistics once the image was loaded into memory, showed an average time to calculate the 27 statistics of 20 ms per image. Threshold adjacency statistics are hence an order of magnitude faster to calculate than either the Haralick or Zernike measures.

In general, segmentation of cell images into cellular and non-cellular regions is a difficult problem. When calculating the average intensity μ of pixels whose intensity is at least 30, the lower bound 30 was chosen as intensities below this value are in general background for the endogenous and transfected image sets. The sensitivity of the predictive accuracy using TAS for different choices of lower bound was tested as follows. As described above, a lower bound of 30 gave a 5-fold cross validation accuracy of 95.2% on the endogenous set. Further tests with a lower bound of 40 gave an accuracy of 94.2%, and a lower bound of 20 gave an accuracy of 96.6%. Tests with the transfected image set yielded similarly small variation (data not shown). Hence, while there is some variation in accuracy, threshold adjacency statistics appear relatively insensitive to the choice of threshold.

A commonly used auto-thresholding scheme is to find a threshold intensity *t*, such that *t *is (approximately) the average of the average intensity of those pixels with intensity less than or equal to *t*, and the average intensity of those pixels with intensity greater than *t *(see ImageJ FAQ [22]). Using such a scheme on the endogenous image set with threshold adjacency statistics gave a 5-fold classification accuracy of 91.6%. Visually examining the selections showed auto-thresholding had had variable success in highlighting the cellular regions of the images. One particular problem was that there was a general trend to under-select cells, that is to miss regions. To compensate for this a lower bound of the auto threshold value minus 15 was tested and gave an accuracy of 93.2%. Hence auto-thresholding, while performing reasonably well, is computationally more expensive and is not as effective as choosing a fixed threshold when applying threshold adjacency statistics. It is possible that another variable thresholding scheme or segmentation algorithm might give better cell region selection results and hence better predictive accuracy with threshold adjacency statistics, but the computational complexity of such a scheme is likely to be high.

Another point to consider is that cell populations may not be heterogeneous due to variations in cell cycle. In preparing the image sets, DAPI images were examined to exclude those cells that were not in interphase. For non-heterogeneous populations an interesting and useful addition could be to apply an automated cell phase predictor such as is described in by Pham et al. in [23] prior to classification. Cells that are not in interphase could then either be excluded or treated separately.

## Conclusion

Threshold adjacency statistics have been shown to be well suited to sub-cellular localization classification, and offer a number of advantages over other image statistics. With a classification accuracy of up to 95% they offer comparable or better accuracy than the Haralick texture measures, while being an order of magnitude faster to calculate. While comparison with previous literature is problematic in that each group has distinct image sets with different sub-cellular classes and varying degrees of automation, threshold adjacency statistics appear at least on par with a reported 92% accuracy previously obtained [19]. Automated region selection and cropping of cells for classification can be exceptionally difficult and computationally expensive, especially when cells are highly confluent. Threshold adjacency statistics require no cropping and are additive, hence giving better statistics the more cells there are in an image. Another advantage is that every image presented is classified. With automated cropping/selection systems a wide range of images are dealt with, and so it is not uncommon to fail to locate a cell within an image because it is relatively faint, or some other criteria. Further, for applications in which speed of calculation is not critical, the use of threshold adjacency statistics in combination with Haralick texture measures give an accuracy of up to 98%. Finally, with 3D and 4D cell imaging become more widespread, new methods are required to distinguish and classify protein localization. While automated classification of 3D sub-cellular localization using image statistics has proved very successful [24], the addition of an extra dimension greatly increases the computational expense, and hence application of threshold adjacency statistics to 3D has the potential to significantly increase classification throughput.

## Methods

### Image data sets collection

An image collection was created for sub-cellular organelles consisting of either or both of two types of sets; one set for which an endogenous protein or feature of the specific organelle was detected with a fluorescent antibody or other probe (10 organelles); and another set for which an epitope- or fluorescence-tagged protein was transiently expressed in the specific organelle and subsequently detected (11 organelles). Each image was accompanied by an additional image of the cells counterstained with the DNA specific dye 4',6-diamidino-2-phenylindole (DAPI), which highlights the location of the nucleus of every cell in the image. In addition, the DAPI image was reviewed to exclude images that contained one or more cells not in interphase. Each organelle set consists of 50 localisation images and 50 DAPI counterstained images, with the exception of the endogenous nuclear which contains only DAPI images. In total, 502 endogenous and 553 transfected localization images were obtained. All images were of fixed HeLa cells, taken at 60× magnification under oil immersion. The images are 8 bit greyscale, 768 by 512 pixels, each containing up to 13 cells. Cropped sample images of each organelle are given in Figures 3 and 4, and the antibodies or probes used are given in Table 1. The complete image set is available for download from the LOCATE website [2].

### Implementation and testing

Image statistics were implemented in C++ within the ASPiC software [20]. The time tests were conducted on a Pentium 4 2.4 GHz machine running Red Hat Enterprise 3.

SVMs were created using the *libsvm *software [25] with a radial basis function (RBF) kernel. Two parameters are required to train the RBF kernel, γ the coefficient of the exponent, and C the penalty term of the error. A grid search was performed to choose those values of γ and C that gave the best 5-fold cross validated performance on each data set. For testing, 5-fold cross validation was utilized. Data is split into 5 equal parts, each part in turn is tested on an SVM trained on the remainder, and the average test set accuracy returned. By splitting data as described above, cross-validation may be used to avoid over-fitting the training data and give an estimate of the prediction error for unseen data, though care does need to be taken [26]. In certain cases, it can be proved that the cross-validation error estimate is an almost unbiased estimate of the true error on unseen data [27]. The 1000 repeated tests of splitting the data sets into 4/5^ths ^for training and 1/5^th ^for testing (Table 1) gave comparable classification accuracies to that of 5-fold cross validation, hence suggesting that over-fitting has not occurred to a significant degree.

## Authors' contributions

NAH designed and tested the threshold adjacency statistics and drafted the manuscript. RSP implemented the software for the threshold adjacency and other statistics described herein. KH created the sub-cellular localization image sets. RDT participated in the design of the study and coordination and helped to draft the manuscript. All authors read and approved the final manuscript.
